# External Oblique Muscle Schwannoma: A Rare Anatomical Presentation

**DOI:** 10.1155/2019/9290821

**Published:** 2019-09-03

**Authors:** Daniel Paramythiotis, Diamantoula Pagkou, Moysis Moysidis, Niki Mantha, Angeliki Cheva, Antonios Michalopoulos

**Affiliations:** ^1^1st Propaedeutic Surgery Department, AHEPA University Hospital of Thessaloniki, Greece; ^2^Pathology Department, Faculty of Medicine, Aristotle University of Thessaloniki, Greece

## Abstract

**Introduction:**

Schwannomas or neurilemmomas are rare, benign, and usually solitary lesions that arise from the nerve sheath. In the majority of cases, these tumors involve the extremities, head, neck, and trunk.

**Case Presentation:**

In the present study, a 52-year-old man presented to our surgical department for the evaluation of a symptomatic lump in his left lateral abdominal wall. CT and MRI confirmed the presence of a cystic mass located between the external and internal oblique muscles. Histopathology and immunohistochemistry confirmed it to be benign schwannoma.

**Discussion:**

Schwannomas have rarely been reported in the abdominal wall. To the best of our knowledge, this is the first case of schwannoma located in the left upper abdominal wall and the fifth case of abdominal wall schwannoma reported according to the reviewed medical literature.

**Conclusion:**

Benign schwannoma should be included in the differential diagnosis of cystic and symptomatic lesions of the abdominal wall. The treatment of choice is surgical excision, and recurrence is extremely rare.

## 1. Introduction

Schwannomas, also known as neurilemmomas or neurinomas, are benign and slow-growing neoplasms originating from Schwann cells of the peripheral nerve sheath. They constitute approximately 5% of all benign soft-tissue tumors. The majority of these tumors are commonly localized in the trunk, head, neck, and extremities [1–3].

Their presentation in the abdominal wall is extremely rare, and to the best of our knowledge, this is the sixth reported case of an abdominal wall benign schwannoma [3–7]. In addition, this is the fourth symptomatic case [3, 6, 7].

The present study reports the case of a 52-year-old man with a schwannoma of the left upper lateral abdominal wall. The patient provided his written informed consent.

## 2. Case Report

We present a case of a 52-year-old man who presented to our outpatient clinic complaining of a painful lump in his left lateral abdominal wall. The patient noticed the development of a small nodule about one year ago, and he also stated that the lump was gradually increasing in size. The patient did not refer any previous abdominal trauma or any further pathologies. Palpation revealed a 4 × 5 cm nodular mass protruding through the left upper lateral abdominal wall. The mass was firm, tender, and not fix to the skin of the abdominal wall.

**Figure 1:**
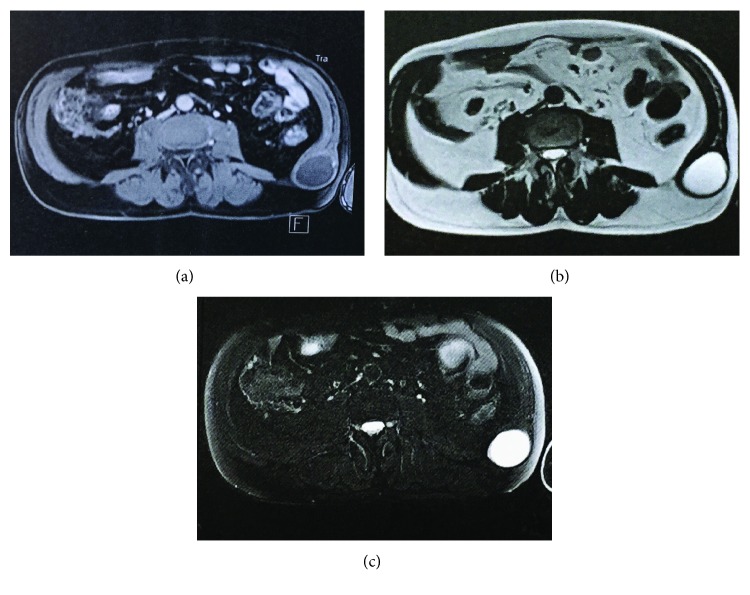
(a) Transverse section of postcontrast T1 MRI abdomen revealed a well-circumscribed mass located between the external and internal oblique muscles. (b) T2W MRI clearly delineates the capsule of the lesion and internal morphology. (c) The lesion is intensely hyperintense on fat-suppressed MRI.

Computed tomography (CT) scan of the abdomen with contrast revealed a 32 × 45 mm cystic mass located between the external and internal oblique muscles (Figure 1(a)). Abdominal Magnetic Resonance Imaging (MRI) demonstrated a well-circumscribed 4 × 5 cm cystic mass, arising in the abdominal wall. The lesion was hyperintense on T2 (Figure 1(b)), and fat-suppressed sequences suggested cystic degeneration (Figure 1(c)). Clinical appearance and radiological findings were not specific for a particular entity. Our differential diagnosis included a chronic haematoma or a cystic tumor of the abdominal wall.

**Figure 2:**
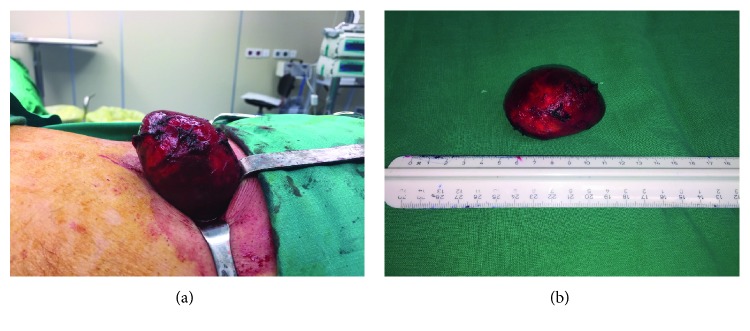
(a) Perioperative image revealing the nodular mass. (b) Image of the resected specimen.

The patient underwent total resection of the mass under local anaesthesia (Figure 2(a)). The choice of local anaesthesia was based on the patient's preference, the small size of the tumor, and the fact that it was located at the abdominal wall. During the operation, the tumor was intermuscular and found between the external and the internal oblique muscle. The mass was not strongly attached to the surrounding tissues and was easily mobilised and excised in healthy margins. He was discharged the following day in excellent clinical condition.

**Figure 3:**
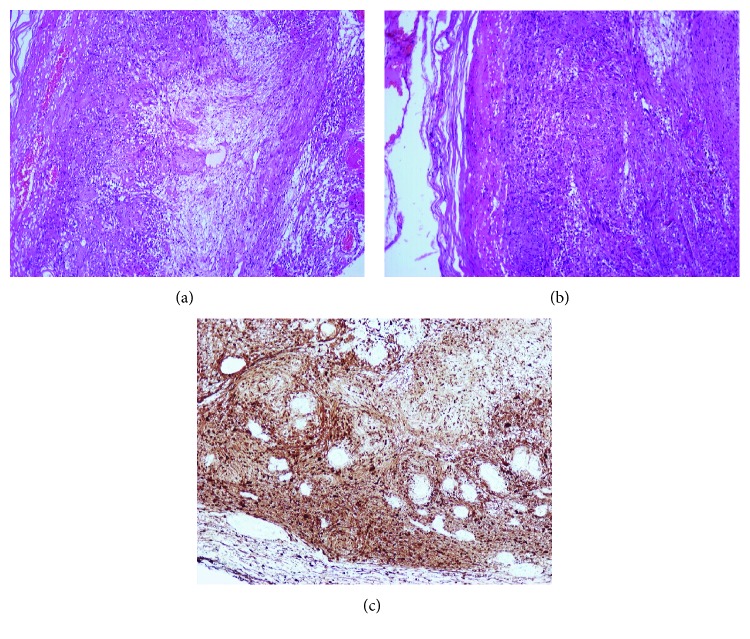
(a, b) Hematoxylin and Eosin stain (H&E) from the tumor wall. Spindled Schwann cells forming hypercellular Antoni A areas with nuclear palisading and Verocay bodies with alternating hypocellular-myxoid area (Antoni B). (c) The tumor cells stained positive for S-100 protein.

Macroscopic examination of the resected specimen revealed a well-circumscribed nodular mass measured 6.5 × 4.5 × 3.5 cm (Figure 2(b)). Histopathology findings were characterized by interlacing bundles of spindle cells of varying cellularity and peripheral lymphoid cuffs (Figures 3(a)–3(b)).

The neoplastic cells were immunoreactive with S-100 protein (Figure 3(c)) and vimentin. The resected margin was reported clear (R0). Postoperative period was uneventful, and one-year follow-up was unremarkable.

## 3. Discussion

Schwannomas are benign neurogenic tumors, originating from Schwann cells, which normally wrap around the axons of the peripheral nerves and can develop anywhere along the peripheral course of the nerve [1, 2, 8]. Schwannomas are usually solitary lesions, and they have a predilection for the trunk, head, neck, and flexures or surfaces of the limbs [1–3]. They have rarely been reported on the abdominal wall [5–7].

Natural history studies consistently show that the overall average patient age at schwannoma presentation is the fourth decade of life, without a significant sex predilection. Schwannomas are usually sporadic, but they can also be associated with genetic disorders such as neurofibromatosis type 2, schwannomatosis, or Carney's complex. Rare descriptions exist of malignant change in long-standing neurilemmomas, usually in patients with an underlying diagnosis of neurofibromatosis. Malignant transformation is extremely rare in isolated lesions [2, 8, 9].

To the best of our knowledge, only five cases of benign schwannoma located in the abdominal wall have been reported in the medical literature at present [3–7]. In the first case [4], a healthy 64-year-old female underwent a whole body CT scan, which revealed an incidental mass in the right iliac fossa. In the second case [5], a 24-year-old female presented with a painless lump in her left abdominal wall. The third study [6] reported the case of a 57-year-old woman presented with localized abdominal pain in her left lower quadrant. Followingly, abdominal computed tomography (CT) scan revealed a 17 × 11 mm mass in her abdominal wall. In the fourth case [7], a 62-year-old woman was admitted for the evaluation of a painful mass in her right iliac fossa. The fifth case [3] reported the case of a 70-year-old man presented with a painless nodule localized in his left abdominal wall. In all of these cases, a benign schwannoma was confirmed histopathologically.

Schwannomas may grow slowly and may be present for months or years without causing symptoms. Symptoms usually appear when they have grown to the point where they are putting pressure to the nerves near them. Symptoms include a visible lump, numbness, muscle weakness, and aching, burning, or sharp pains. In our case, due to the location of the mass below the external oblique muscle, pain may result from the compression and irritation of the thoracoabdominal and subcostal nerves.

On CT and MRI, schwannomas appear as well-circumscribed cystic masses of low to intermediate signal intensity on T1-weighted sequences and high signal intensity on T2-weighted sequences, caused by a predominance of hypercellular Antoni A type tissue [10, 11].

Macroscopically, schwannomas are presented as well-circumscribed masses. Histologically, they are characterized by hypercellularity (Antoni type A) and hypocellular-myxoid (Antoni type B) areas. Nuclear palisading around fibrillary process (Verocay bodies) is often seen in cellular areas [9].

By immunohistochemistry, schwannomas show diffuse and strong expression of S-100 protein. Recent markers frequently positive in schwannomas include podoplanin, calretinin, and SOX10 [12].

## 4. Conclusions

Although schwannoma of the abdomen wall is extremely rare, it should be considered in the differential diagnosis of cystic and painful lesions of the abdominal wall. Complete surgical excision is the treatment of choice for these lesions, and malignant transformation is extremely rare. Recurrence is unlikely after complete resection.

## Additional Points

*Highlights*. (i) Schwannomas are rare, benign, and usually solitary lesions that arise from the nerve sheath. (ii) Schwannomas are rarely located in the abdominal wall. (iii) Most neurilemmomas are asymptomatic, if not, they present with pain, numbness, and muscle weakness. (iv) If excised completely, schwannomas seldomly recur.
